# Simulação no Ensino de Medicina Cardiovascular: Inovação, Segurança e Treinamento Baseado em Competências

**DOI:** 10.36660/abc.20260233

**Published:** 2026-07-15

**Authors:** André Luiz Lisboa Cordeiro, Tiago Veltri Ormastroni da Trindade, Rinaldo Antunes Barros

**Affiliations:** 1 Centro Universitário de Excelência Feira de Santana BA Brasil Centro Universitário de Excelência, Feira de Santana, BA – Brasil; 2 Escola Bahiana de Medicina e Saúde Pública Salvador BA Brasil Escola Bahiana de Medicina e Saúde Pública, Salvador, BA – Brasil

**Keywords:** Treinamento por Simulação, Cardiologia, Segurança do Paciente, Educação Médica, Competência Profissional

A incorporação de simuladores no ensino da medicina cardiovascular representa uma das transformações mais significativas na formação médica contemporânea. Em um cenário marcado por alta complexidade clínica, rápida evolução tecnológica e tomada de decisões em tempo real, a simulação surge como uma estratégia pedagógica capaz de integrar conhecimento teórico, habilidades técnicas e competências não técnicas de forma estruturada e segura.^1,2^ No entanto, apesar de sua crescente adoção, a educação baseada em simulação deve ser interpretada dentro do contexto das evidências científicas atuais, que ainda apresentam limitações importantes, particularmente em relação ao seu impacto direto nos desfechos centrados no paciente.

A cardiologia contemporânea, caracterizada por rápidos avanços em terapias farmacológicas, procedimentos intervencionistas complexos e exames de imagem cardíaca sofisticados, exige que os profissionais não apenas adquiram novas habilidades, mas também mantenham proficiência contínua. Nesse contexto de rápida evolução e crescentes demandas por segurança e eficácia, a simulação se posiciona como uma importante aliada. Contudo, seu papel deve ser compreendido como complementar, e não substitutivo, da exposição clínica real. Essas necessidades são reforçadas pelas recém-estabelecidas Diretrizes Curriculares Nacionais para o Curso de Graduação em Medicina (DCNs 2025), que defendem explicitamente a oferta de "ambientes de aprendizagem protegidos, controlados e tecnicamente adequados, como laboratórios de habilidades e simulação clínica", visando à segurança do paciente e à análise de erros como ferramenta pedagógica, enfatizando também que o treinamento clínico em ambientes reais de saúde deve permanecer predominante ao longo da formação médica.^3^ Essa visão alinha-se à experiência já consolidada de programas que formam multiplicadores de simulação, essenciais para a educação continuada de profissionais de saúde no âmbito do SUS e para a formação de estudantes e residentes.^4^

A cardiologia exige domínio de procedimentos invasivos, interpretação rápida de dados clínicos e hemodinâmicos, bem como comunicação eficaz em equipes multidisciplinares. Tradicionalmente, essas competências eram adquiridas predominantemente à beira do leito, em um modelo de aprendizado oportunista. No entanto, limitações éticas, preocupações com a segurança do paciente e menor exposição a casos complexos impulsionaram a adoção de metodologias alternativas, entre as quais a simulação ocupa uma posição de destaque.^5^ A simulação clínica surge, portanto, como uma ferramenta importante para a prática segura. A oportunidade de praticar repetidamente procedimentos complexos e manejar crises cardiovasculares de alta criticidade e baixa frequência, como dissecções aórticas agudas, punção transeptal, intervenções estruturais (por exemplo, TAVI) ou falhas de dispositivos implantáveis, em um ambiente simulado, é fundamental.^4,6^ Contudo, a heterogeneidade das modalidades de simulação (baixa vs. alta fidelidade, modelos virtuais vs. híbridos) e a variabilidade nos desenhos dos estudos limitam a generalização dos resultados e representam desafios à validade externa e à transferibilidade para a prática clínica real. Esta abordagem não só melhora a competência técnica, como também, conforme enfatizado pelas novas DCNs (2025), garante a oferta de "ambientes de aprendizagem protegidos, controlados e tecnicamente adequados" que permitam a "identificação e análise de erros como instrumento pedagógico para o aperfeiçoamento profissional".^3^ Ao minimizar o risco de eventos adversos e erros na área da saúde,^7^ esta metodologia alinha-se com um imperativo ético na formação, permitindo aos alunos "vivenciar situações de erros e acertos, relatar e discutir sistematicamente" tais ocorrências para "aprimorar esses sistemas por meio de uma compreensão abrangente da falibilidade humana".^4^

Uma das principais vantagens da simulação é sua capacidade de promover a aprendizagem integrada. Os alunos são desafiados não apenas a executar técnicas, mas também a interpretar sinais clínicos, tomar decisões sob pressão e coordenar ações em equipe. Evidências demonstram que o treinamento baseado em simulação está associado a um melhor desempenho clínico, maior retenção de conhecimento e desenvolvimento de habilidades de liderança e comunicação.^8,9^ No entanto, é importante destacar que a maior parte das evidências disponíveis se concentra em resultados educacionais intermediários, com dados limitados e heterogêneos sobre seu impacto em desfechos clínicos concretos, como morbidade, mortalidade ou redução de eventos adversos. O escopo da aprendizagem em simulação vai além da mera proficiência técnica. As novas DCNs (2025) afirmam explicitamente a necessidade de os médicos "exercerem liderança colaborativa em ambientes interprofissionais" e "demonstrarem empatia, escuta ativa, comunicação eficaz e capacidade de trabalho colaborativo".^3^ Nesse contexto, a simulação proporciona um ambiente seguro e controlado para o desenvolvimento dessas competências não técnicas.^4^

Fundamentalmente, a eficácia da educação baseada em simulação depende muito da qualidade da instrução e, particularmente, do debriefing estruturado. Além do seu impacto educacional, a simulação contribui para a padronização do ensino e da avaliação. Cenários estruturados permitem a avaliação objetiva de competências por meio de listas de verificação e métricas de desempenho.^8^ O debriefing sistematizado, considerado o elemento central da simulação, estimula a reflexão crítica e a aprendizagem com os erros.^2,9^ Evidências atuais demonstram consistentemente que a maior parte da aprendizagem em simulação ocorre durante a fase de debriefing e que atividades de simulação conduzidas sem um debriefing eficaz têm valor educacional limitado. É dentro desse processo interativo e reflexivo que a experiência prática se transforma em aprendizagem consolidada, fomentando o pensamento crítico e o raciocínio clínico.^4,9^ Para esse fim, as novas DCNs (2025) destacam a necessidade de estruturas de desenvolvimento docente, como o "Centro de Apoio Pedagógico e Experiência Docente (NAPED)",^3^ garantindo que os facilitadores sejam adequadamente treinados.

Apesar dos benefícios amplamente documentados, ainda existem desafios. O alto custo dos simuladores de alta fidelidade, a necessidade de treinamento do corpo docente e a integração efetiva da simulação nos currículos médicos ainda limitam sua disseminação, especialmente em países de baixa e média renda.^10^ No entanto, a inovação tecnológica e a criatividade oferecem caminhos promissores para superar essas barreiras. A criação e o uso de simuladores de baixo custo e artesanais têm se mostrado alternativas acessíveis.^4^ Além disso, a combinação de diferentes níveis de fidelidade em modelos híbridos e a promoção de redes colaborativas entre instituições de ensino e hospitais podem democratizar o acesso. Ao mesmo tempo, a integração de tecnologias emergentes, como a inteligência artificial, deve ser abordada criticamente. Os riscos potenciais incluem viés algorítmico, dependência da tomada de decisão automatizada, redução do desenvolvimento do raciocínio clínico e desafios relacionados à qualidade dos dados e ao uso ético. Portanto, a incorporação dessas tecnologias deve seguir os princípios do uso ético, crítico e responsável, conforme recomendado pelas diretrizes educacionais atuais.^11,12^

Além disso, é preciso reconhecer os importantes desafios práticos relacionados à implementação, incluindo a integração curricular, a distribuição da carga de trabalho, o treinamento do corpo docente e a adaptação à realidade heterogênea das instituições de ensino, principalmente em contextos com recursos limitados. Pesquisas futuras devem explorar mais a fundo a relação entre o treinamento baseado em simulação e os resultados na área da saúde, tais como a redução de eventos adversos e melhorias na segurança do paciente em cardiologia.

Em resumo, o uso de simuladores no ensino da medicina cardiovascular representa um avanço pedagógico consistente, alinhado às demandas da prática clínica moderna. Ao possibilitar um treinamento seguro, integrado e reproduzível, a simulação contribui para a formação de profissionais mais bem preparados para lidar com a complexidade e a criticidade inerentes à cardiologia contemporânea. Contudo, seu papel deve ser compreendido como complementar ao treinamento clínico em situações reais, e não como um substituto. Portanto, é imprescindível que a comunidade acadêmica e os gestores de saúde reconheçam a simulação não apenas como um método de ensino, mas como um investimento estratégico e ético.^3,4^ Uma abordagem mais crítica e equilibrada, que reconheça tanto seus pontos fortes quanto suas limitações, será essencial para fortalecer sua credibilidade científica e otimizar sua implementação na educação médica.

## Data Availability

Todo o conjunto de dados que dá suporte aos resultados deste estudo está disponível mediante solicitação ao autor correspondente.
