# Supplementary material for: Simulação no Ensino de Medicina Cardiovascular: Inovação, Segurança e Treinamento Baseado em Competências
Source: Arq Bras Cardiol. 2026 Jul 15;123(6):e20260233. [Article in Portuguese] doi: 10.36660/abc.20260233 (PMC13399271; doi:10.36660/abc.20260233)
Supplement: Translation

# Translation: Simulation in Cardiovascular Medicine Education: Innovation, Safety, and Competency-Based Training

**DOI:** 10.36660/abc.20260233i

**Authors:** André Luiz Lisboa Cordeiro, Tiago Veltri Ormastroni da Trindade, Rinaldo Antunes Barros

**Affiliations:** 1 Feira de Santana BA Brazil Centro Universitário de Excelência, Feira de Santana, BA – Brazil; 2 Salvador BA Brazil Escola Bahiana de Medicina e Saúde Pública, Salvador, BA – Brazil

**Keywords:** Simulation Training, Cardiology, Patient Safety, Medical Education, Professional Competence

The incorporation of simulators into cardiovascular medicine education represents one of the most significant transformations in contemporary medical training. In a scenario marked by high clinical complexity, rapid technological evolution, and time-dependent decision-making, simulation emerges as a pedagogical strategy capable of integrating theoretical knowledge, technical skills, and non-technical competencies in a structured and safe manner.^1,2^ However, despite its growing adoption, simulation-based education should be interpreted within the context of its current evidence base, which still presents important limitations, particularly regarding its direct impact on patient-centered outcomes.

Contemporary cardiology, characterized by rapid advances in pharmacological therapies, complex interventional procedures, and sophisticated cardiac imaging, requires professionals not only to acquire new skills but also to maintain continuous proficiency. In this context of rapid evolution and increasing demands for safety and effectiveness, simulation positions itself as an important ally. Nevertheless, its role should be understood as complementary rather than substitutive to real clinical exposure. These needs are reinforced by the newly established National Curricular Guidelines for the Undergraduate Medical Course (DCNs 2025), which explicitly advocate the provision of "protected, controlled, and technically adequate learning environments, such as skills laboratories and clinical simulation," aiming at patient safety and the analysis of error as a pedagogical tool, while also emphasizing that clinical training in real healthcare environments must remain predominant throughout medical education.^3^ This vision aligns with the already consolidated experience of programs that train simulation multipliers, which are essential for the continuing education of health professionals within the SUS and for the training of students and residents.^4^

Cardiology requires mastery of invasive procedures, rapid interpretation of clinical and hemodynamic data, as well as effective communication within multidisciplinary teams. Traditionally, these competencies were predominantly acquired at the bedside, in an opportunistic learning model. However, ethical limitations, concerns about patient safety, and reduced exposure to complex cases have driven the adoption of alternative methodologies, among which simulation holds a prominent position.^5^ Clinical simulation thus emerges as an important tool for safe practice. The opportunity to repeatedly practice complex procedures and manage high-criticality, low-frequency cardiovascular crises, such as acute aortic dissections, transseptal puncture, structural interventions (e.g., TAVI), or failures of implantable devices, in a simulated environment is fundamental.^4,6^ Nonetheless, the heterogeneity of simulation modalities (low vs. high fidelity, virtual vs. hybrid models) and variability in study designs limit the generalizability of findings and pose challenges to external validity and transferability to real-world clinical practice. This approach not only enhances technical competence but, as emphasized by the new DCNs (2025), ensures the provision of "protected, controlled, and technically adequate learning environments" that allow the "identification and analysis of error as a pedagogical instrument for professional improvement".^3^ By minimizing the risk of adverse events and healthcare errors,^7^ this methodology aligns with an ethical imperative in training, enabling students to "experience situations of errors and successes, systematically report and discuss" such occurrences to "improve these systems through a comprehensive understanding of human fallibility".^4^

One of the main advantages of simulation is its ability to promote integrated learning. Students are challenged not only to perform techniques but also to interpret clinical signs, make decisions under pressure, and coordinate team actions. Evidence demonstrates that simulation-based training is associated with improved clinical performance, greater knowledge retention, and the development of leadership and communication skills.^8,9^ However, it is important to highlight that most available evidence is focused on intermediate educational outcomes, with limited and heterogeneous data regarding its impact on hard clinical outcomes such as morbidity, mortality, or reduction of adverse events. The scope of learning in simulation extends beyond mere technical proficiency. The new DCNs (2025) explicitly state the need for physicians to "exercise collaborative leadership in interprofessional environments" and to "demonstrate empathy, qualified listening, effective communication, and the ability to work collaboratively".^3^ In this context, simulation provides a safe and controlled environment for developing these non-technical competencies.^4^

Crucially, the effectiveness of simulation-based education is highly dependent on the quality of instruction and, particularly, on structured debriefing. Beyond its educational impact, simulation contributes to the standardization of teaching and assessment. Structured scenarios allow objective evaluation of competencies through checklists and performance metrics.^8^ Systematized debriefing, considered the central element of simulation, stimulates critical reflection and learning from error.^2,9^ Current evidence consistently demonstrates that the majority of learning in simulation occurs during the debriefing phase, and that simulation activities conducted without effective debriefing have limited educational value. It is within this interactive, reflective process that practical experience is transformed into consolidated learning, fostering critical thinking and clinical reasoning.^4,9^ To this end, the new DCNs (2025) highlight the need for faculty development structures such as the "Center for Pedagogical Support and Teaching Experience (NAPED)",^3^ ensuring that facilitators are adequately trained.

Despite the widely documented benefits, challenges remain. The high cost of high-fidelity simulators, the need for faculty training, and the effective integration of simulation into medical curricula still limit its dissemination, especially in middle- and low-income countries.^10^ However, technological innovation and creativity offer promising pathways to overcome these barriers. The creation and use of low-cost and handcrafted simulators have proven to be accessible alternatives.^4^ Furthermore, combining different levels of fidelity in hybrid models and promoting collaborative networks among educational institutions and hospitals can democratize access. At the same time, the integration of emerging technologies such as artificial intelligence should be approached critically. Potential risks include algorithmic bias, dependence on automated decision-making, reduced development of clinical reasoning, and challenges related to data quality and ethical use. Therefore, the incorporation of such technologies must follow principles of ethical, critical, and responsible use, as recommended by current educational guidelines.^11,12^

Additionally, important practical challenges related to implementation should be acknowledged, including curricular integration, workload distribution, faculty training, and adaptation to the heterogeneous reality of educational institutions, particularly in resource-limited settings. Future research should more robustly explore the relationship between simulation-based training and healthcare outcomes, such as the reduction of adverse events and improvements in patient safety in cardiology.

In summary, the use of simulators in cardiovascular medicine education represents a consistent pedagogical advancement aligned with the demands of modern clinical practice. By enabling safe, integrated, and reproducible training, simulation contributes to the preparation of professionals better equipped to deal with the complexity and criticality inherent in contemporary cardiology. Nevertheless, its role should be understood as complementary to real-world clinical training, rather than a replacement. It is therefore imperative that the academic community and healthcare managers recognize simulation not merely as a teaching method but as a strategic and ethical investment.^3,4^ A more critical and balanced approach, acknowledging both its strengths and limitations, will be essential to strengthen its scientific credibility and optimize its implementation in medical education.

## Data Availability

All datasets supporting the results of this study are available upon request from the corresponding author.
